# Exploring the interactions between metabolic dysfunction-associated fatty liver disease and micronutrients: from molecular mechanisms to clinical applications

**DOI:** 10.3389/fnut.2024.1344924

**Published:** 2024-03-14

**Authors:** Yuan Liu, Xiang Qin, Tianzhu Chen, Mengyao Chen, Liyan Wu, Beihui He

**Affiliations:** ^1^The First Affiliated Hospital of Zhejiang Chinese Medical University (Zhejiang Provincial Hospital of Chinese Medicine), Hangzhou, China; ^2^Department of Gastroenterology, Tongde Hospital of Zhejiang Province, Hangzhou, China

**Keywords:** metabolic (dysfunction)-associated fatty liver disease, vitamins, minerals, nutritional assessment, therapeutic strategy

## Abstract

Metabolic (dysfunction)-associated fatty liver disease (MAFLD) has emerged as a significant global health concern, representing a major cause of liver disease worldwide. This condition spans a spectrum of histopathologic stages, beginning with simple fatty liver (MAFL), characterized by over 5% fat accumulation, and advancing to metabolic (dysfunction)-associated steatohepatitis, potentially leading to hepatocellular carcinoma. Despite extensive research, there remains a substantial gap in effective therapeutic interventions. This condition’s progression is closely tied to micronutrient levels, crucial for biological functions like antioxidant activities and immune efficiency. The levels of these micronutrients exhibit considerable variability among individuals with MAFLD. Moreover, the extent of deficiency in these nutrients can vary significantly throughout the different stages of MAFLD, with disease progression potentially exacerbating these deficiencies. This review focuses on the role of micronutrients, particularly vitamins A, D, E, and minerals like iron, copper, selenium, and zinc, in MAFLD’s pathophysiology. It highlights how alterations in the homeostasis of these micronutrients are intricately linked to the pathophysiological processes of MAFLD. Concurrently, this review endeavors to harness the existing evidence to propose novel therapeutic strategies targeting these vitamins and minerals in MAFLD management and offers new insights into disease mechanisms and treatment opportunities in MAFLD.

## 1 Introduction

Non-alcoholic fatty liver disease (NAFLD) represents a spectrum of liver disorders, ranging from simple steatosis to more severe conditions like steatohepatitis with fibrosis, and ultimately, cirrhosis. Recognizing its association with hepatic steatosis, obesity, T2DM, and hypertriglyceridemia, NAFLD has been renamed Metabolic (Dysfunction)-Associated Fatty Liver Disease (MAFLD), highlighting its metabolic underpinnings (1–3).

MAFLD can lead to hepatocellular carcinoma in its more advanced stages, a malignancy known for its high mortality rate. Recent epidemiological studies reveal that MAFLD’s global prevalence has reached approximately 30% (4), and this trend shows no signs of abating. Most MAFLD patients initially have a benign condition, MAFL, with over 5% of hepatocytes containing lipid droplets (5). However, 20–30% progress to metabolic (dysfunction)-associated steatohepatitis (6, 7), characterized by significant steatosis, inflammation, and cellular ballooning, primarily in the liver’s alveolar zone 3 (8). Alarmingly, up to 38% of MASH patients with fibrosis may develop cirrhosis, and 2.4–12.8% of these individuals are at risk of hepatocellular carcinoma (HCC) (7). Both cirrhosis and hepatocellular carcinoma linked to MAFLD are associated with poor prognoses, highlighting the urgency for timely and effective management strategies in MAFLD patients.

Vitamins and minerals, essential micronutrients predominantly sourced from our diet, play a crucial role in normal body functioning through their antioxidant properties, enzyme activities, and immune system modulation (9). Recent research has brought to light the significant role of certain trace elements, particularly vitamins A, D, E, and minerals like iron, copper, selenium, and zinc. This article delves into their involvement in immune-inflammatory and metabolic processes (10, 11). The destabilization of these micronutrients has been linked to a variety of metabolic diseases (12), including MAFLD (13). Globally, vitamin and mineral deficiencies are widespread (14), and MAFLD patients frequently face similar challenges. These deficiencies are often tied to the dietary choices (15) of the individuals and a reduction in vitamin production due to altered intestinal flora (16). Despite the prevalence of MAFLD, current medical treatments remain inadequate. However, observations of micronutrient imbalances in MAFLD patients and animal models (17), along with the improvements seen in targeted therapies, open up new avenues for treating this condition. The ability of vitamins and trace minerals to positively impact the mechanisms at the core of MAFLD offers promising prospects for its pharmacological treatment (18, 19). This insight, focusing on correcting micronutrient imbalances, could pave the way for innovative strategies in managing and potentially mitigating the progression of MAFLD.

**Figure 1:**
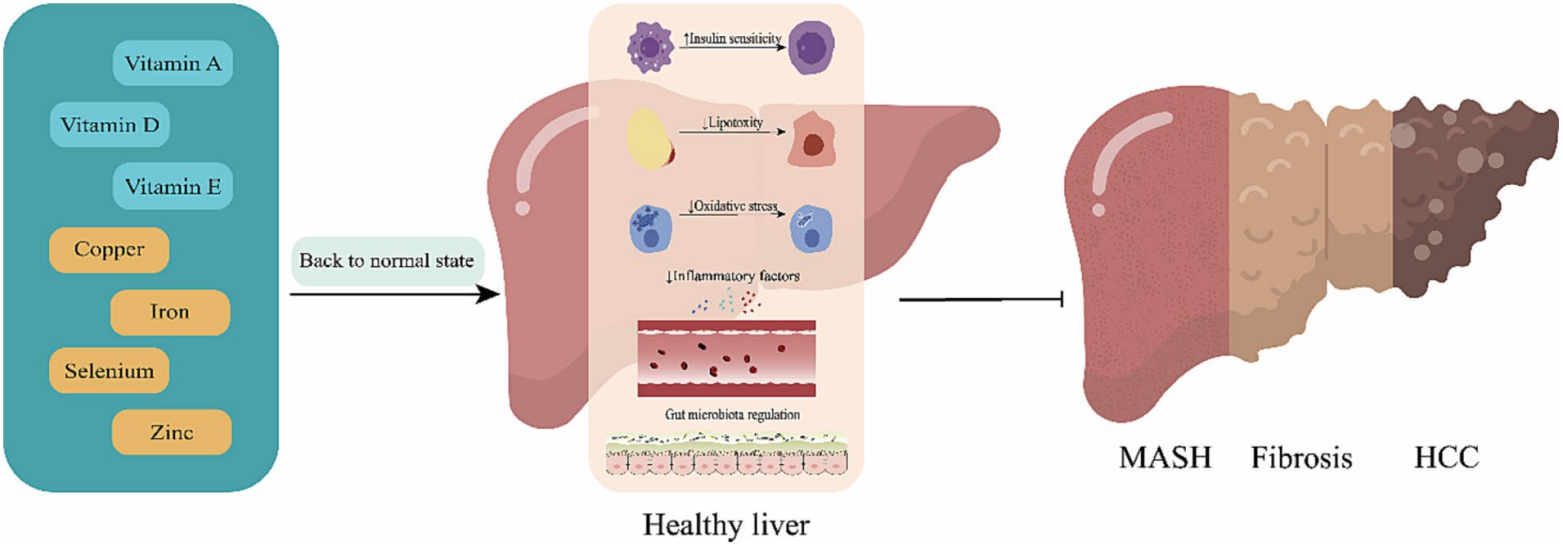
Mechanisms of action for the effect of micronutrients. Replenishment of deficient micronutrients plays a pivotal role in reducing the risk and progression of metabolic (dysfunction)-associated fatty liver disease (MAFLD). By restoring these essential nutrients to optimal levels, there is a marked improvement in insulin sensitivity, a reduction in lipotoxicity, a decrease in inflammatory mediators, and a regulation of the intestinal microbiota. These changes collectively contribute to slowing the progression from metabolic (dysfunction)-associated fatty liver (MAFL) to metabolic (dysfunction)-associated steatohepatitis, fibrosis, and potentially hepatocellular carcinoma.

The review aims to provide the latest summary on the pathophysiologic pathways linking micronutrients to the development of MAFLD and to focus on new data from clinical trials exploring the safety and efficacy of vitamin and mineral supplementation on liver outcomes in patients with MAFLD (See Figure 1).

## 2 Pathogenesis of MAFLD and current therapeutics

### 2.1 Pathogenesis of MAFLD

The pathogenesis of MAFLD is not well defined, and the “multi-hit theory” is more widely recognized. MAFLD is a complex disease characterized by interactions between the environment and the susceptible polygenic host background that determine the phenotype and progression of the disease, MBOAT7, and other variants in the genes are strongly and consistently associated with MAFLD (20). On this basis, modern high-fat diet and unhealthy lifestyle habits act as triggers for impaired hepatic fat metabolism, hepatocellular fat accumulation producing lipotoxicity (21), endoplasmic reticulum stress (22), increased synthesis of reactive oxygen species, synthesis of adipokines, activation of inflammatory cells and release of inflammatory factors triggering intrahepatic inflammation (23), disruption of hepatic homeostasis, and comorbid insulin resistance (24). Gut microecological changes (25), accelerating the transformation of MAFL to MASH, liver fibrosis and cirrhosis (See Table 1).

**Table 1:** Effect of vitamins and minerals in MAFLD.

Author (reference)	Treatment and control	Experimental model	Treatment dosage and administration	Findings
Tang et al. (26)	Treatment: retinoic acid receptor β2 agonist(AC261066) Negative controls:- Positive controls:-	High-fat diet (HFD) induced wild-type (wt) male C57BL/6 mice mouse	3 mg/100 mL drinking water, oral for 2 months	↓mRNA increases inPklr, Fasn, Thrsp., and Chchd6 ↓transcript and protein levels of KHK
Trasino et al. (27)	Treatment: retinoic acid receptor β2 agonist(AC261066) Negative control: RARγ agonist (CD1530) Positive control: no treatment	High-fat diet (HFD) induced Wild type (wt) male C57BL/6 mice	15 IU/vitamin A-acetate/gram, oral for 3 months	↓hepatic steatosis and oxidative stress ↓expression of pro-inflammatory mediators ↓hepatic stellate cell (HSC) activation ↓kupffer TGF-β1 expression
Zarei et al. (28)	Treatment: atRA Negative control:- Positive control: -	High-fat diet (HFD) induced male New Zealand rabbits	5 mg/kg/day, oral for 30 days	↓liver steatosis ↓liver oxidative agents ↑total antioxidant capacity
Kim et al. (29)	Treatment: atRA Negative control: - Positive control: -	WD-fed C57BL/6 mice	Corn oil containing atRA (15 mg/kg/day), oral for 7 days	↓adiposity in brown fat
Berry et al. (30)	Treatment: atRA Negative control: - Positive control: -	High-fat/high-sucrose diet C57BL/6Ntac mice	Subcutaneously implanted with an RA pellet or mock pelleted by using a 10-gauge precision trochar	↑weight loss ↑insulin responsiveness
Tsuchiya et al. (31)	Treatment: ATRA Negative control: - Positive control:-	High-fat, high-fructose diet-induced C57BL/6 J mice	50 mg/kg ATRA, oral for 4 weeks	↓insulin resistance
Li et al. (18)	Treatment: 1,25 (15)2D3 Negative control: - Positive control: -	High-fat diet (HFD) induced male C57BL/6 mice	2.5 ng/g, three times per week for 4 weeks, i.p.	↓liver inflammation regulated lipid metabolism
Dabbaghmanesh et al. (32)	Treatment: cholecalciferol & calcitriol Negative control: placebo Positive control: no treatment	MAFLD patient	50,000 U vitamin D3 pearl/week for 3 months, oral or0.25 mg calcitriol pearl/day for 3 months, oral	↓serumalkaline phosphatase and GGT
Wenclewska et al. (33)	Treatment: cholecalciferol Negative control: no treatment Positive control: no treatment	Metabolic Disorder patients	2000 International Unit (11) cholecalciferol/day oral for three months	↓oxidative stress ↓insulin resistance ↑metabolic profile
El Amrousy et al. (34)	Treatment: vitamin D Negative control: placebo Positive control: no treatment	100 children with biopsy-proven MAFLD	2000 IU/day orally for 6 months	↓hepatic steatosis ↓lobular inflammation
Mosca et al. (19)	Treatment: vitamin E & hydroxytyrosol Negative control: placebo Positive control: no treatment	Children with MAFLD	3.75 mg of hydroxytyrosol plus 5 mg of Vitamin E/day, oral for 16 weeks	↓systemic inflammation ↓oxidative stress
Scorletti et al. (35)	Treatment: vitamin E Negative control: no treatment Positive control: no treatment	MAFLD patients	Not for sure	↓overall mortality ↓risk of MAFLD
Vilar-Gomez et al. (36)	Treatment: vitamin E Negative control: no treatment Positive control: no treatment	MASH patients	800 international units/day of vitamin E for ≥2 years	↑clinical outcomes
Podszun et al. (37)	Treatment:vitamin E Negative control: no treatment Positive control: no treatment	MAFLD patients	200–800 IU/d, oral for 24 weeks	↓oxidative stress
Doboszewskaet al. (38)	Treatment: Zinc Negative controls: Zinc-deficient (ZnD)diet Positive controls: Zinc-adequate (ZnA) diet	Male Sprague-Dawley rats	50 mg Zn/kg or 3 mg Zn/kg, for 4 or 6 weeks	↓oxidative damage ↓pro-inflammatory status
Ma et al. (39)	Treatment: high-iron (HI) diets Negative controls: low-iron (LI) diets Positive controls: -	Male db/db mice	High-iron (HI) diets (1,000 mg/kg chow) or low-iron (LI) diets (12 mg/kg), oral for 9 weeks	↑Gluconeogenesis ↓Lipogenesis ↑Insulin resistance
Fujiwara et al. (40)	Treatment: high-iron Negative controls: Western diet Positive controls: Western diet + high-iron	Male F344/DuCrlCrlj rats	6% of blending iron citrate (FeC6H5O7・5H2O), oral for 26 weeks	↑serum triglyceride and cholesterol ↑hepatic inflammation
Wang et al. (41)	Treatment: Se-enriched spirulina Negative controls: normal diet with Se-enriched spirulina Positive controls: HFD with Se-enriched spirulina	High-fat diet (HFD) induced C57BL/6 mice	Se content 0.45 mg/kg, oral 12 weeks	↓hepatic injury and insulin resistance ↓fat accumulation & expression of lipogenic genes
Xu et al. (42)	Treatment: sodium selenate, Negative controls: - Positive controls: -	Male APP/PS1 transgenic mice	12 μg/mL sodium selenate, oral for 2 months	↓insulin resistance
Zhang et al. (43)	Treatment: Se Negative controls: - Positive controls: -	free fatty acid (FFA) induced primary rat hepatocytes	0.1 μM Se, cell cultivation	↓oxidative stress ↓apoptosis
Miyataet al. (6)	Treatment: Selenoneine Negative controls: - Positive controls: -	Fxr-null mice	0.3 mg Se/kg selenoneine-containing diet oral for 4 months	↓hepatocellular injury ↓hepatic steatosis

### 2.2 Current therapeutics

Current treatment strategies for Metabolic Dysfunction-Associated Fatty Liver Disease (MAFLD) are limited in effectiveness. Clinical guidelines primarily advocate for lifestyle interventions (44) and, in cases where obesity has advanced significantly, bariatric surgery (45) to facilitate weight loss. For lean MAFLD patients, the recommended approach is lifestyle modification coupled with a reduction in fructose and sugar-sweetened beverages, aiming for a modest weight loss of 3–5% (46). Other pharmacological treatments, such as metformin, thiazolidinediones, and liraglutide, are generally reserved for patients with concurrent diabetes mellitus. However, their efficacy specifically for MAFLD is not conclusively proven, and they have shown potential side effects or unintended results in animal studies (47).

Research into MAFLD patients’ micronutrient levels reveals complex interactions and trends. Vitamins A, E, Zinc, and Copper are often reduced, while Vitamin D varies and Iron increases. These micronutrients interact within MAFLD, complicating disease understanding and progression. This interplay presents challenges yet offers new therapeutic opportunities. Current research focuses on understanding these interactions to develop targeted MAFLD treatments, marking a shift towards more effective management approaches.

## 3 Vitamins and MAFLD

### 3.1 Vitamins deficiency status in MAFLD patients

Vitamin deficiency is a global health issue with widespread impact (48). In metabolic diseases related to obesity, most vitamins are found to be deficient (49). Specifically, in MAFLD, the primary vitamins affected are the fat-soluble ones: A, D, and E. MAFLD patients, often consuming diets low in nutrients, rich in high-fat meats/proteins, and high in sodium (50), are prone to lower levels of these vitamins without additional supplementation. Furthermore, alterations in the intestinal microecology significantly influence vitamin absorption, contributing to the vitamin deficiencies observed in MAFLD patients (51, 52). Additionally, vitamin deficiencies play a role in low-intensity inflammation, exacerbated by the release of inflammatory adipokines from adipose tissue, which further aggravates the condition of MAFLD patients. This complex interplay underscores the importance of addressing vitamin deficiencies in managing and improving the health outcomes for those with MAFLD (49).

### 3.2 Role of vitamins in pathogenesis of MAFLD

#### 3.2.1 Vitamin A and MAFLD

Vitamin A, essential for various physiological functions in the human body, relies exclusively on dietary intake. Its primary active form, retinoic acid (RA), plays a pivotal role by binding to retinoic acid receptors to facilitate biological signal transduction (53). Normally, vitamin A, being fat-soluble, is stored in hepatic stellate cells (54, 55). In patients with MAFLD, circulating concentrations of retinoic acid are observed to be lower (56). There is a notable correlation between diminished levels of Vitamin A and the severity of hepatic fibrosis, as well as an increase in liver-related mortality (57). Furthermore, in patients with metabolic (dysfunction)-associated Steatohepatitis (MASH), high expression of hepatic AKR1B10 is linked to reduced hepatic retinoid levels, exacerbating the progression from MASH to Hepatocellular Carcinoma (HCC) (58). This highlights the critical role of Vitamin A in liver health and its potential implications in the progression of liver diseases.

Vitamin A contributes to the management of MAFLD through various mechanisms, including the modulation of lipid metabolism (26, 30, 59), antioxidant effects (28, 60), anti-inflammatory properties (27), and enhancing insulin sensitivity (30, 31). Notably, the retinoic acid receptor β2 agonist AC261066 has been shown to induce changes in the transcriptome and metabolome of hepatocytes (26), reduce the TGF-β1 inflammatory response in Kupffer cells, and alleviate liver fibrosis (27, 61). Dietary supplementation with all-trans retinoic acid (ATRA) notably improved insulin sensitivity in MAFLD model mice (C57BL/6J) (31). Additionally, ATRA acts on the retinoic acid receptor (62) to decrease PPAR-γ2 expression, thereby reducing fat accumulation in the liver (29).

Despite these promising findings, the clinical application of vitamin A in MAFLD treatment is constrained by its narrow therapeutic window and the limited number of clinical trials. This highlights the need for further research to fully understand and harness Vitamin A’s potential in MAFLD treatment while ensuring safety and efficacy in human applications.

#### 3.2.2 Vitamin D and MAFLD

Vitamin D, primarily produced in the skin via sunlight exposure, is vital for both skeletal and extra-skeletal health. Clinical guidelines recommend keeping human serum 25(OH)D levels above 50 nmoL/L. Despite this, about 7% of the global population has vitamin D levels below this threshold (63). Studies indicate that vitamin D deficiency is nonlinearly linked to increased MAFLD severity and higher all-cause mortality (64). Lower 25(OH) vitamin D levels are associated with increased MAFLD prevalence and liver fibrosis (65), while higher levels reduce fibrosis risk in MAFLD patients (66).

In a Western diet rat model, Vitamin D deficiency exacerbated MAFLD, potentially via toll-like receptor activation and endotoxin exposure (67). This deficiency also caused insulin resistance, increased hepcidin expression, and heightened inflammation and oxidative stress genes. Key to this severity in vitamin D-deficient MAFLD patients might be the activation of MAPK and NF-κB pathways (68).

In the realm of treating MAFLD with vitamin D, significant strides have been made. Studies across various regions (69, 70) and populations (71, 72) have shown that increased vitamin D levels may help prevent MAFLD. Different dosages of vitamin D exhibit varying degrees of improvement in MAFLD (33, 73).

Vitamin D induces autophagy (18) in mice, primarily by upregulating ATG16L1, thereby inhibiting the p53 pathway to prevent hepatocyte senescence and apoptosis (74). It also reduces inflammation via the enterohepatic axis, underscoring the importance of timely supplementation (75). Phototherapy-enhanced active vitamin D3 in mice mitigates hepatocyte apoptosis, inflammation, fibrosis, and insulin/leptin resistance caused by a CDAA diet (76). Additionally, vitamin D treatment curbs MAFLD induced by a high-fat diet (HFD), involving gut microbiota (77) and metabolic regulation (78), and modulates lipid metabolism through the PPARa signaling pathway (79). Vitamin D-regulated miRNAs are implicated in MAFLD pathogenesis, though more research is needed (80). It also exhibits antifibrotic effects by countering TGF-β signaling in hepatic stellate cells (81).

Contrastingly, some studies have found no correlation between plasma vitamin D levels and insulin resistance, hepatic fat accumulation, or MASH severity (82–85). Similarly, randomized trials using vitamin D supplements for MAFLD treatment have not consistently shown benefits (73, 86). Polymorphisms in the Vitamin D receptor gene could explain the varied outcomes observed. While Vitamin D ameliorates liver damage in MAFLD, early expression of its receptor in MAFLD patients’ livers and decreased lipid accumulation in mice lacking this receptor gene point to its intricate involvement in MAFLD’s development and progression (87).

These findings highlight vitamin D’s potential in MAFLD treatment but also reveal its multifaceted and context-dependent nature. The genetics and epigenetics of MAFLD may influence vitamin D’s regulatory mechanisms, necessitating further research to elucidate these intricate relationships.

#### 3.2.3 Vitamin E and MAFLD

Vitamin E, currently the only medication recommended by guidelines for treating MASH, is valued for its antioxidant and anti-inflammatory properties (88, 89). It has been observed that patients with MAFLD often have reduced serum levels of both vitamin E and A (90). In a non-randomized, propensity score-adjusted study, a daily intake of 800 IU of vitamin E was associated with significant reductions in total mortality and hepatic decompensation in patients with MASH-induced bridging fibrosis and cirrhosis, both in diabetic and non-diabetic individuals (36). Moreover, vitamin E has been effective in lowering AST and ALT levels in adult patients with MAFLD (91). It inhibits oxidative stress, which reduces *de novo* lipogenesis (DNL) and intrahepatic triglyceride (IHTG) accumulation, thereby disrupting the cycle between oxidative stress and the MAFLD process (92). Histological improvements in MAFLD patients have also been noted with vitamin E treatment, demonstrating its therapeutic potential (92–95).

However, the effectiveness of vitamin E in altering the histological course of MASH in patients with Type 2 Diabetes Mellitus (T2DM) has not been significant (96). Additionally, its use is limited in the treatment of common comorbidities associated with MAFLD (96). While vitamin E shows promise in MAFLD treatment, its role and efficacy may vary depending on specific patient conditions and comorbidities, indicating the need for a nuanced approach in its clinical application.

## 4 Minerals and MAFLD

### 4.1 Minerals deficiency status in MAFLD patients

Mineral deficiencies are widely acknowledged as a significant public health issue worldwide, often leading to increased susceptibility to infections. By replenishing these deficient trace minerals to their recommended levels, we can enhance immune function, bolster resistance to infection, and facilitate quicker recovery from such illnesses. While epidemiological data on the connection between trace mineral deficiencies and the onset and advancement of MAFLD are scant, the role of inflammation as a key contributor to MAFLD, coupled with the dietary habits commonly observed in individuals with MAFLD, suggests a potential close link between these mineral deficiencies and the disease’s development and progression.

### 4.2 Role of minerals deficiency in the process of MAFLD progression

#### 4.2.1 Major minerals

Calcium, phosphorus, and magnesium, as major minerals, play important roles in MAFLD. These minerals are key factors in the inflammatory processes related to MAFLD, participating in signaling mechanisms, hepatocyte injury and regeneration, and the regulation of inflammatory factors (62, 97–99). The intricate roles of these major minerals in the human body and their specific associations with MAFLD have been extensively discussed in other reviews (100) and studies (101), and thus fall outside the primary focus of this paper. Instead, this review concentrates on trace minerals, including zinc, iron, copper, and selenium, exploring their relationship with MAFLD and their impact on the progression and management of the disease.

#### 4.2.2 Zinc and MAFLD

Zinc, a crucial trace element, plays vital roles in antioxidant, anti-inflammatory, and anti-apoptotic functions in the human body (102, 103). The risk of zinc deficiency increases with age (104). There is growing evidence linking zinc deficiency to the development of MAFLD (105, 106). In patients with biopsy-proven MAFLD, a J-shaped correlation exists between serum zinc levels and the severity of hepatic necroinflammation (107, 108). Serum zinc deficiency, commonly associated with oxidative stress, endoplasmic reticulum stress, apoptosis, and inflammation, has been noted in MAFLD mouse models (109, 110).

Furthermore, zinc supplementation has been shown to alleviate disorders in lipid and glucose metabolism caused by high-fat diets (111). In diet-induced MAFLD mice, zinc supplementation not only improves liver weight and morphology but also helps prevent hepatic failure (112).

Recent studies reveal zinc’s mechanisms in improving MAFLD: in mouse models, PLZF, relying on SIRT1, regulates hepatic lipid and glucose homeostasis (113). The HDAC3/β-catenin pathway promotes lipolysis and inhibits adipogenesis (114). ZHX2 activation of PTEN protects against MASH progression (115). Zinc alpha2 glycoprotein in hepatocytes impacts triglyceride accumulation and key gene expressions (116). The ADA/XO/UA pathway and caspase 3 signaling show potential in liver rescue (116). Zinc oxide nanoparticles in mice reduce hepatic steatosis via the AMPK axis (117), while the Zn2+/MTF-1/PPARa pathway aids in reducing lipid deposition (118). These findings collectively highlight zinc’s multifaceted role in addressing various aspects of MAFLD pathogenesis and progression.

Despite that, the specific studies and recommended zinc dosages for clinical treatment of MAFLD still require further exploration and validation.

#### 4.2.3 Iron and MAFLD

The increasingly recognized causal link between iron overload and the progression of MAFLD (119, 120) suggests that dietary iron overload may worsen inflammation and lipid metabolism disorders, akin to human dietary iron overload syndrome (DIOS) (40). Hyperferritinemia the main manifestation of disturbed iron homeostasis often portends more severe metabolic dysfunction and liver injury (121, 122). In the hypoxic intestinal environment, HIF-2alpha plays a crucial role in regulating iron absorption by affecting the DMT1 gene (123). Abnormal iron-induced hepcidin release, influenced by natural genetic variants may enhance iron absorption (124). Additionally, excess free fatty acids (FFAs) disrupt hepatic iron metabolism, encouraging iron uptake via IRP1 and TfR-1 (125). Iron overload contributes to ferroptosis, initiating inflammation in nonalcoholic steatohepatitis and leading to oxidative DNA damage (126, 127). This condition can be exacerbated by a high-fat diet, which aggravates lipid metabolism disorders, hepatic injury, and oxidative stress (128). Iron-containing extracellular vesicles from hepatocytes induce liver steatosis and fibrosis in mice on a Western diet, causing iron deficiency in hepatocytes and overload in hepatic stellate cells (129). The complex interaction between gut microflora and the host not only impacts MAFLD progression but also influences iron balance (130, 131). This situation results in a detrimental cycle where iron overload increases lipid deposition through oxidative stress-induced mitochondrial dysfunction and activation of the HIF1α-PPARγ pathway (129, 132).

While numerous studies have indicated that bloodletting to address iron overload can improve insulin resistance in patients with MAFLD and hyperferritinemia (133–135), other findings suggest that lowering ferritin through phlebotomy does not necessarily improve liver enzymes, liver fat, or insulin resistance in MAFLD patients (136). This discrepancy highlights the need for more detailed research to unravel these complex interactions and effects.

#### 4.2.4 Copper and MAFLD

Copper, a vital cofactor in numerous physiological redox reactions, has a complex relationship with MAFLD. *In vivo* bioluminescence imaging has shown copper deficiency in a mouse model of MAFLD (137). Concurrently, both hair and hepatic copper concentrations in MAFLD patients are significantly lower and correlate with increased hepatic steatosis, MASH severity, and metabolite alterations (138). Limiting copper intake in mice has been shown to induce hepatic steatosis and insulin resistance, leading to the development of MAFLD (17, 139).

Research exploring the link between copper and lipid metabolism indicates a negative correlation (140). Restoration of intrahepatic copper, achieved by down-regulating copper cyanin, enhances lipolysis through the assembly of copper-loaded SCO1-LKB1-AMPK complexes, showing improvements in MAFLD conditions in mice (141). A case-control study found that high levels of copper significantly improved MAFL in males, highlighting copper’s protective role in MAFLD treatment (140, 142).

However, studies also point out the harmful effects of copper overload on lipid metabolism (143, 144) and increased MAFLD risk and severity (145). Moreover, there is a noticeable gap in clinical research exploring the relationship between copper and MAFLD, and the potential toxicology of copper also warrants special attention. Despite these complexities, the intricate link between copper and MAFLD presents a potential avenue for breakthroughs in MAFLD treatment.

#### 4.2.5 Selenium and MAFLD

Selenium, a crucial micronutrient, plays diverse roles in the human body, including antioxidant activities, cancer prevention, and immunomodulation, thanks to its structural and enzymatic functions (146–148). It also has significant implications in metabolic diseases (149). The relationship between selenium and MAFLD is complex and appears to be dose-dependent (150).

Studies have shown that lower blood selenium levels are associated with a higher incidence of advanced liver fibrosis (151). Conversely, higher blood selenium levels (above ~130 μg/L) have been positively correlated with both MAFLD and ghrelin, indicating a dose–response relationship (150, 152). In experimental settings, selenium supplementation in MAFLD mice models has demonstrated beneficial effects, such as mitigating hepatic injury, reducing oxidative stress, lowering insulin resistance, and decreasing inflammation (6, 41, 43, 153).

However, the use of selenium in MAFLD treatment necessitates careful consideration of its delicate balance between therapeutic efficacy and toxicity. Determining the appropriate dosage of selenium is critical and remains a subject of ongoing research and debate in the context of MAFLD treatment. This nuanced understanding of selenium’s role underscores the importance of precise dosing in its potential application as a therapeutic agent for MAFLD.

### 4.3 Relationship between micronutrients in MAFLD

While individual trace elements’ roles in MAFLD have been detailed, research on their complex interrelationships is scarce. Zinc and selenium have been linked to reduced cardiovascular risk (109), and Vitamin D and zinc both enhance immune function (154). Additionally, copper and ascorbic acid can interfere with non-heme iron absorption (155). These findings indicate a delicate balance among trace elements, crucial for maintaining overall body homeostasis.

## 5 Conclusion and outlook

In this review, we examine recent research on the impact of various vitamins and trace minerals on MAFLD. Most studies suggest that deficiencies in vitamins and minerals negatively affect MAFLD. Timely and appropriate supplementation could aid in disease recovery or slow its progression, potentially improving patient prognosis. However, there are also contrasting views or skepticism regarding the causal link between these deficiencies and MAFLD, an aspect this review critically explores.

Currently, there’s no unified approach to the pharmacological treatment of MAFLD. Lifestyle interventions and bariatric surgery have shown relative effectiveness, but their success is often limited by patient compliance and eligibility criteria. Hence, their widespread application among MAFLD patients is restricted. The search for effective drugs targeting MAFLD’s pathogenesis continues. Vitamins and minerals, crucial in regulating oxidative stress, inflammation, and lipid metabolism, offer promising directions for MAFLD treatment. The need for a drug that can improve the course and prognosis of MAFLD, provided in the necessary amounts for normal body function, is urgent.

Given the complex pathophysiology of MAFLD, the effectiveness of single-agent treatments observed in various studies suggests that individualized combination regimens might be necessary for optimal management of MAFLD. This review seeks to shed light on these multifaceted approaches and the potential of vitamins and minerals in the treatment landscape of MAFLD.

## Author contributions

YL: Writing – original draft. XQ: Writing – original draft. TC: Writing – original draft. MC: Writing – original draft. LW: Writing – review & editing. BH: Supervision, Writing – review & editing.
